# Isolation and characterization of a new population of nasal surface macrophages and their susceptibility to PRRSV-1 subtype 1 (LV) and subtype 3 (Lena)

**DOI:** 10.1186/s13567-020-00751-7

**Published:** 2020-02-24

**Authors:** Dayoung Oh, Jiexiong Xie, Nathalie Vanderheijden, Hans J. Nauwynck

**Affiliations:** Department of Virology, Immunology, and Parasitology, Faculty of Veterinary Medicine, Ghent University, Salisburylaan 133, 9820 Merelbeke, Belgium

## Abstract

Sialoadhesin (Sn) and CD163 have been recognized as two important mediators for porcine reproductive and respiratory syndrome virus (PRRSV) in host macrophages. Recently, it has been demonstrated that the highly virulent Lena strain has a wider macrophage tropism than the low virulent LV strain in the nasal mucosa. Not only CD163^+^Sn^+^ macrophages are infected by Lena but also CD163^+^Sn^−^ macrophages. This suggests that an alternative receptor exists for binding and internalization of PRRSV Lena in the CD163^+^Sn^−^ macrophages. Further investigation to find the new entry receptor was hampered by the difficulty of isolating these macrophages from the nasal mucosa. In the present study, a new population of CD163^+^Sn^−^ cells has been identified that is specifically localized in the nasal lamina propria and can be isolated by an intranasal digestion approach. Isolated nasal cells were characterized using specific cell markers and their susceptibility to two different PRRSV-1 strains (LV and Lena) was tested. Upon digestion, 3.2% (flow cytometry)—6.4% (confocal microscopy) of the nasal cells were identified as CD163^+^ and all (99.7%) of these CD163^+^ cells were Sn^−^. These CD163^+^Sn^−^ cells, designated as “nasal surface macrophages”, showed a 4.9 times higher susceptibility to the Lena strain than to the LV strain. Furthermore, the Lena-inoculated cell cultures showed an upregulation of CD163. These results showed that our new cell isolation system is ideal for the further functional and phenotypical analysis of the new population of nasal surface macrophages and further research on the molecular pathogenesis of PRRSV in the nose.

## Introduction

Porcine reproductive and respiratory syndrome virus (PRRSV) is one of the most economically important pathogens in the swine industry, causing reproductive failure in sows and respiratory disorders in piglets [1]. It belongs to the family *Arteriviridae*, the order of the *Nidovirales* [2]. Further classification placed PRRSV in the genus *Betaarterivirus*, and 40% of genetic variation divides PRRSV into PRRSV-1 (subgenus *Eurpobartevirus*) and PRRSV-2 (subgenus *Ampobartevirus*) [3]. PRRSV-1 is divided into three subtypes with different distributions in Europe and Asia. PRRSV-2 is common in Asia and the Americas [4]. In the 2000s, highly pathogenic PRRSV-1 subtype 3 strains emerged in Eastern Europe [5].

One of the major routes of PRRSV transmission is via nose–nose contact and the air [6]. Epithelial cells in the nasal airway commonly serve as the primary entry site for many viruses. The lamina propria is located underneath the epithelium. This is a special connective tissue consisting of a complex network of fibers, filaments, and immune cells such as lymphocytes and macrophages [7]. Lymphocytes and macrophages in the epithelial cell layer and the lamina propria are also important targets of viruses [8]. Several viruses replicate in these cells to traverse the epithelium barrier, migrate through the lamina propria and end up in the blood circulation [9, 10]. PRRSV is one of these “smart” viruses. It uses resident macrophages in the mucosa to replicate and induce a viremia [11].

PRRSV has a restricted cell tropism for cells of the monocyte-macrophage lineage. Several membrane receptors or cellular proteins such as heparin sulfate, sialoadhesin (also known as Sn, siglec-1 and CD169), siglec-10, DC-SIGN (also known as CD209), CD163, CD151, vimentin, and non-muscle myosin heavy chain 9 (MYH9) have been identified as mediators for PRRSV entry into permissive cells [12–15]. Scavenger receptor CD163 is considered as an indispensable mediator for PRRSV infection because CD163 expression in non-permissive cell lines makes them susceptible to PRRSV infection and CD163 knockout pigs are resistant to infection with PRRSV [16–21]. Among these mediators, the intriguing interplay between siglecs and CD163 has been extensively studied. Previously, a PRRSV entry model has been described based on the use of two main entry mediators, siglecs and CD163. Siglecs mediate virus attachment and internalization, and CD163 coordinates viral disassembly [13, 14, 17, 22–24]. However, previous studies from our laboratory showed that the highly virulent PRRSV-1 subtype 3 Lena strain has a wider cell tropism than PRRSV-1 subtype 1 Lelystad strain. This strain is not only able to infect CD163^+^Sn^+^ but also CD163^+^Sn^−^ macrophages in the nasal mucosa [11, 25]. Moreover, Prather et al. [26] has reported PRRSV-2 infection in the Sn knockout pigs. These observations suggest that Sn is not the only attachment and internalization receptor for PRRSV and an alternative entry mediator together with the disassembly mediator CD163 might be responsible for this highly virulent PRRSV strain infection of the CD163^+^Sn^−^ nasal macrophages. These cells are also siglec-10 negative [13]. Therefore, the entry mediator in the CD163^+^Sn^−^ nasal macrophages remains to be identified.

The aim of this study is to develop an in vitro CD163^+^Sn^−^ nasal macrophage isolation system, which will form the basis for further identification of alternative PRRSV entry mediators in the Sn^−^ cells. In this study, we first analyzed the distribution of CD163^+^ macrophages in the entire porcine nasal mucosa and the Sn expression in these cells. Next, a new digestion system was developed to specifically isolate the CD163^+^Sn^−^ nasal macrophages and to test their susceptibility to two prototype PRRSV-1 strains, LV (subtype 1) and Lena (subtype 3).

## Materials and methods

### Animals

The study was performed with the nasal mucosa from 8 to 10-week old healthy conventional pigs from a PRRSV-negative farm. A total of 6 pigs was used in this study. Three pigs were used for the immunofluorescence staining of the nasal tissue cryosections, and three pigs were euthanized for the whole nose digestion, cell characterization, and PRRSV-1 inoculation experiments.

### Nasal mucosa collection

**Figure 1:**
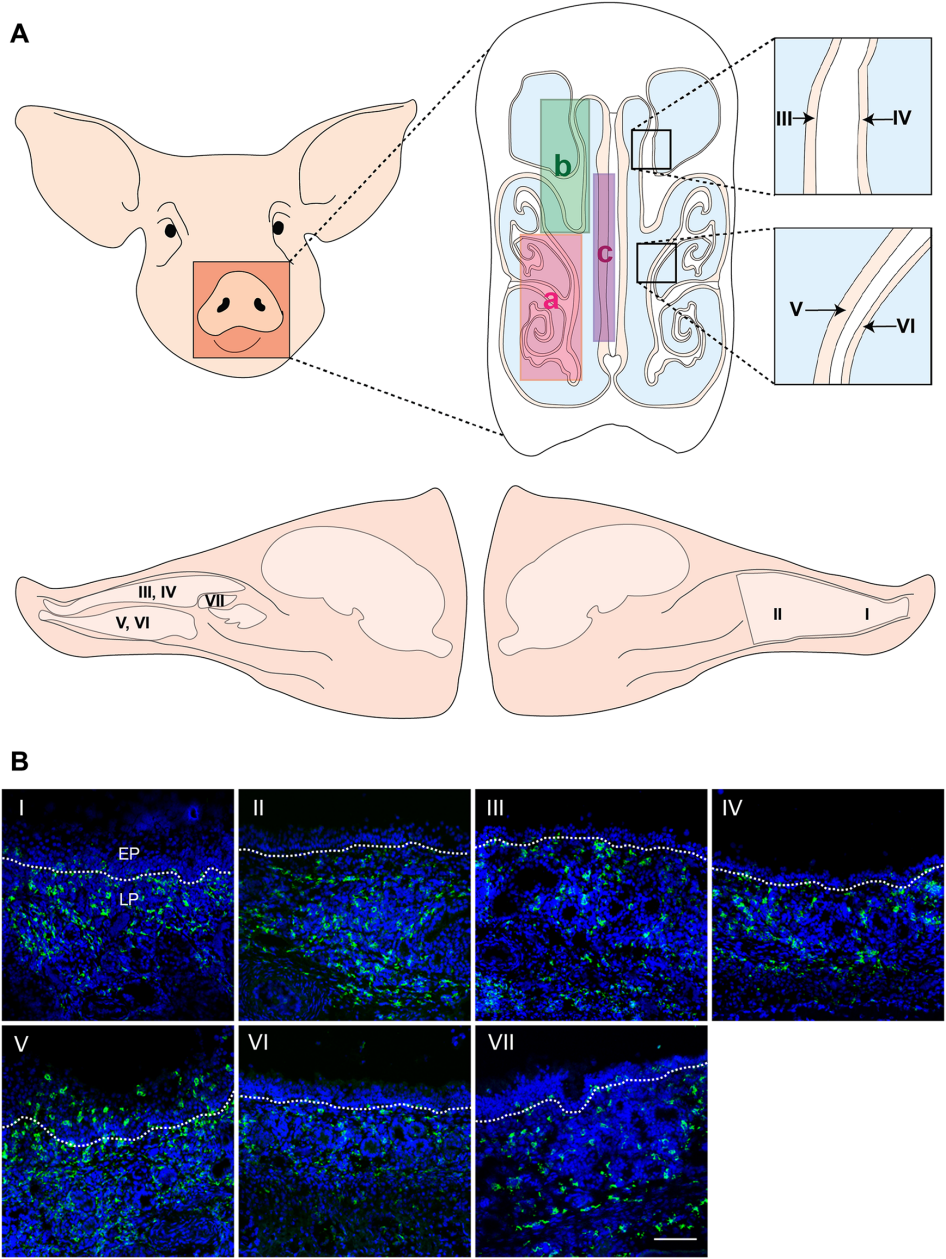
**Distribution of CD163 positive cells in the porcine nasal mucosa. A** Anatomy of the porcine nose: (a) ventral turbinate, (b) dorsal turbinate, (c) septum. Colors represent cartilage (white), airway (blue), mucosa (incarnadine). **B** Sections of porcine nasal tissues were subjected to immunofluorescent staining for CD163 (green): (I) anterior nasal septum, (II) posterior nasal septum, (III) medial side of the dorsal nasal turbinate, (IV) lateral side of the dorsal nasal turbinate, (V) medial side of the ventral nasal turbinate, (VI) lateral side of the ventral nasal turbinate, and (VII) middle nasal turbinate. Nuclei were counterstained with Hoechst (blue). White lines indicate the border between the mucosal epithelium and the lamina propria. EP: epithelium, LP: lamina propria. Scale bar: 100 µm.

The pigs were euthanized with 12.5 mg/kg body weight pentobarbital (Kela, Hoogstraten, Belgium). After exsanguination, the head of the pig was cut off from the carcass. The facial skin was stripped from the head, and the head was sawed sagittally. The nasal septum, dorsal turbinates, middle turbinates, and ventral turbinates including cartilage and bone were collected from the nose (Figure 1A). Subsequently, tissues were embedded in methylcellulose medium (ThermoFisher GmbH, Kandel, Germany) and frozen at −70 °C.

### Analysis of the nasal macrophage distribution by immunofluorescence (IF) staining and confocal microscopy

Nine µm cryosections of the methocel-embedded frozen tissue samples were made with a trimming interval of 100 µm between each section. Sections were made using a cryostat at −20 °C and loaded onto 3-aminopropyltriethoxysilane-coated (Sigma-Aldrich, St. Louis, MO, USA) glass slides. Tissue sections were then fixed in 4% paraformaldehyde for 15 min at 4 °C. The fixed sections were washed in PBS and subsequently permeabilized in 0.1% Triton-X diluted in PBS for 10 min at room temperature (RT). Afterwards, the sections were washed in PBS.

To identify the distribution of CD163^+^ macrophages in the nasal mucosa, incubation of 1 h at 37 °C was performed with a mouse monoclonal antibody (mAb) against porcine CD163 (clone 2A10/11, Bio-Rad, Oxford, UK), followed by incubation with FITC-labeled goat anti-mouse IgG1 secondary antibody (1:500, Invitrogen, Eugene, OR, USA) (Table 1).

**Table 1:** **Antibodies used for immunofluorescence staining and flow cytometry**

Primary antibodies	Clone	Isotype	Working dilution	Supplier
Anti-CD163 mAb	2A10/11	IgG1	1:200	Bio-rad
Anti-CD163 pAb	Polyclonal	IgG	1:200	R&D Systems
Anti-Sn mAb	41D3	IgG1	1:50	[27]
Anti-Sn mAb	26B2	IgG2b	1:30	[29]
Anti-MHCII mAb	MSA3	IgG2a	1:200	Kingfisher Biotech
Anti-CD14 mAb	MIL2	IgG2b	1:100	[28]
Anti-CD1c mAb	L161	IgG1	1:50	Biolegend
Anti-cytokeratin mAb	AE1/AE3	IgG1	1:50	Dako
Anti-vimentin mAb	V9	IgG1	1:50	Bio-rad
Anti-PRRSV nucleocapsid protein mAb	13E2	IgG2a	1:50	[32]
Anti-PCV2 Cap mAb	12E12	IgG2a	1:50	[30]
Anti-PRV gB mAb	1C11	IgG2b	1:100	[31]
Anti-PRV gD mAb	13D12	IgG1	1:50	[31]

mAb: monoclonal antibody, pAb: polyclonal antibody.

To identify the Sn positive and negative cells in the CD163 positive and negative cell populations in the nasal mucosa, a double IF staining was performed using goat polyclonal antibody (pAb) against human CD163 (R&D Systems, Mineapolis, MN, USA) and mouse monoclonal antibody (mAb) against porcine sialoadhesin (Sn) (clone 41D3) [27] (Table 1). For the additional characterization of the macrophages in the nasal mucosa, a frozen ventral turbinate section was stained by a triple immunofluorescence with a mouse mAb against porcine Sn (clone 41D3) and a goat pAb against human CD163 together with a mouse mAb against porcine MHCII (clone MSA3, Kingfisher Biotech, St. Paul, MN, USA) or a mouse mAb against porcine CD14 (clone MIL2) [28]; or by a triple immunofluorescence with a mouse mAb against human Sn (clone 26B2) [29] and a goat pAb against human CD163 together with a mouse mAb against human CD1c (clone L161, Biolegend, San Diego, CA, USA) (Table 1).

Primary antibodies were diluted in PBS with 10% rabbit serum and incubated for 1 h at 37 °C, followed by incubation with Alexa Fluor 594 conjugated rabbit anti-goat IgG secondary antibody (1:200, Invitrogen). Afterwards, non-specific binding sites were blocked with 10% negative goat serum for 30 min at 37 °C. The sections were subsequently incubated with FITC-labeled goat anti-mouse IgG1 antibody. For the MHCII, CD14 and CD1c staining, after 1 h incubation with those primary antibodies diluted in PBS with 10% rabbit serum, sections were incubated for 1 h at 37 °C with a rabbit anti-goat IgG Alexa Fluor 647 (1:300, Invitrogen). Afterwards, non-specific binding sites were blocked with 10% goat serum for 30 min at 37 °C. Subsequently, the sections were incubated with either a goat anti-mouse IgG2a Alexa Fluor 594 (1:500, Invitrogen) and a goat anti-mouse IgG1 FITC (1:500) or a goat anti-mouse IgG2b Alexa Fluor 594 (1:200, Invitrogen) and a goat anti-mouse IgG1 FITC for 1 h at 37 °C. A mouse mAb against PCV2 Cap (clone 12E12) [30], a mouse mAb against pseudorabies virus gB (clone 1C11) and a mouse mAb against pseudorabies virus gD (clone 13D12) [31] were used as isotype matched non-specific control (Table 1). Nuclei were counterstained with Hoechst 33242 (10 μg/mL, Invitrogen). Slides were mounted with glycerol-DABCO and analyzed using a TCS SPE confocal system (Leica Microsystems GmbH, Wetzlar, Germany). Since we were interested in macrophages in the sub-epithelium and the upper lamina propria, images with 175 µm depth under the nasal epithelium were taken. The 175 µm was based on the width of a picture taken with a 10× ocular lens and a 63× objective. Ten images per section were randomly taken. The number of CD163 positive cells and total number of cells from each picture were counted and transformed into a percentage.

### Whole nose digestion

The heads of 10-week old conventional pigs were cut off from the carcass after euthanasia with 12.5 mg/kg body weight pentobarbital and exsanguination. After removing the facial skin, both nostrils were closed by suturing (Supramid white, SMI, St. Vith, Belgium) and clamping. The head was fixed upside down with a clamp fixed on a stand. Sterile silicone tubes were inserted into the nasal cavity through the nasopharynx. The nose was washed three times with DPBS (Gibco, Paisley, UK) supplemented with 1 mM ethylene diamine tetra-acetic acid (EDTA) (VWR International, Leuven, Belgium), 0.05 mg/mL gentamicin (Gibco), 0.1 mg/mL streptomycin (Gibco), 100 U/mL penicillin (Gibco) to remove the nasal discharge. Two 20 mL syringes (Romed Holland, CH Wilnis, Netherlands) filled with an enzyme mix [DPBS supplemented with 220 U/mL collagenase type IV (Gibco), 1.4 mg/mL Pronase (Roche Diagnostics GmbH, Mannheim, Germany), 0.1 mg/mL DNase I (Roche Diagnostics GmbH), 2.5 mM d-glucose (VWR International), 1 mM sodium pyruvate (Gibco), 1% non-essential amino acids (Gibco), 0.05 mg/mL gentamicin (Gibco), 0.1 mg/mL streptomycin (Gibco), 100 U/mL penicillin (Gibco)] were connected to the silicone tubes and the enzyme mix was injected into the nasal cavity. In order to selectively isolate CD163^+^Sn^−^ cells located in the upper lamina propria, digestion was carried out at low temperature (whole head on ice) for 72 h. To dissociate the cells from the nasal tissues more effectively, the enzyme mix in the nasal cavity was circulated by using an up-and-down action of the piston 50 times every 3 h. After 24 h and 48 h, the enzyme mix with dissociated cells was collected and a fresh enzyme mix was injected to isolate CD163^+^Sn^−^ cells located deeper in the lamina propria. The enzyme mix with dissociated cells was collected at the 72 h endpoint. Primary cells were passed through a 40 µm cell strainer (VWR international, Radnor, PA, USA) to obtain a uniform single cell suspension. Isolated cells were washed with DPBS supplemented with 1 mM EDTA by centrifugation at 400 × *g* for 10 min at 4 °C. Red blood cells were lysed with erythrocyte lysis buffer (10 mM NaHCO_3_, 155 mM NH_4_Cl, and 10 mM EDTA) (Figure 4). The total number of cells and the viability was determined by trypan blue staining. Then, the cells were directly used for cell characterization and PRRSV-1 infection experiments. After the digestion, the heads were sawed sagittally. The nasal septum, dorsal turbinates, and ventral turbinates were collected from the nose for a double IF staining using goat pAb against human CD163 and mouse mAb against human cytokeratin (clone AE1/AE3, Dako, Carpinteria, CA, USA) to confirm whether the epithelium was removed by the enzyme digestion.

### Characterization of the isolated nasal cells at different digestion times

Dissociated cells from the nasal tissues after 24 h, 48 h, and 72 h digestion were collected. Cells were washed in cold DPBS containing 2% fetal calf serum (FCS) (Sigma-Aldrich), then harvested on slides by cytospinning at 600 × *g* at RT for 8 min (CytoSpin 3, Thermo Shandon, Cheshire, UK). Subsequently, the cells on slides were fixed with 100% methanol for 10 min at −20 °C. To identify the cell types of the primary nasal cell population isolated at each time point, a double IF staining was carried out. Cells were incubated with goat pAb against human CD163 together with mouse mAb against porcine Sn (41D3) or mouse mAb against human cytokeratin (AE1/AE3) or mouse mAb against porcine vimentin (clone V9, Bio-rad) or isotype-matched irrelevant mouse mAb against PRV gD (13D12) for 1 h at 37 °C (Table 1). Subsequently, cells were washed and incubated for 1 h at 37 °C with rabbit anti-goat IgG Alexa Fluor 594 and goat anti-mouse IgG1 FITC; non-specific binding sites were blocked with negative rabbit and goat sera. After washing, nuclei were counterstained with Hoechst 33342. The number of total cells, double-positive cells, and single-positive cells were counted and calculated as percentage by confocal microscopy.

### Flow cytometric analysis of the nasal cells collected after 72 h digestion

One million isolated primary nasal cells were collected on a 96-well plate for each experimental condition. Cells were washed twice in RPMI 1640 (Gibco) containing 1 mM EDTA and 1% FCS. To identify the cell viability, LIVE/DEAD™ Fixable Far Red Dead Cell Stain Kit (Invitrogen) was used according to the manufacturer’s instructions. For the cytoplasm staining, nasal cells were fixed in 4% paraformaldehyde for 15 min on ice and washed with PBS. Afterwards, the cells were permeabilized in 0.1% Triton-X for 10 min on ice. After washing, the cells on each well were subsequently incubated with primary antibodies [mouse mAb against porcine CD163 (2A10), mouse mAb against porcine Sn (41D3), mouse mAb against human cytokeratin (AE1/AE3), mouse mAb against porcine vimentin (V9), or isotype-matched irrelevant mouse mAb against PRV gD (13D12)]. Incubation was performed in the presence of 1 mM EDTA and 1% FCS for 30 min on ice. After washing, cells were incubated with FITC-labeled goat anti-mouse IgG1 secondary antibody in the presence of 1 mM EDTA and 1% FCS for 30 min on ice in the dark. Flow cytometry was performed with a CytoFLEX (Beckman Coulter, Pasadena, CA, USA). 10 000 events were recorded, 1000 events were displayed and doublets were excluded with a gating strategy based on forward light scatter and sideward light scatter. Acquired data were analyzed by CytExpert 2.3 software (Beckman Coulter).

### Virus inoculation of the isolated nasal cells

Isolated primary nasal cells were cultured in complete RPMI 1640 supplemented with 10% FCS, 1 mM sodium pyruvate, 1% non-essential amino acids, 0.05 mg/mL gentamycin, 0.1 mg/mL streptomycin, and 100 U/mL penicillin. Two PRRSV strains were used in this study: LV [prototype PRRSV-1, subtype 1, 13 passages in porcine alveolar macrophages (PAM)] and Lena (prototype PRRSV-1, subtype 3, 4 passages in PAM). Primary nasal cells were seeded at 2 × 10^5^ cells/mL in a 24 well plate (1 mL/well) and after 2 h of incubation, they were inoculated with LV and Lena at a multiplicity of infection (MOI) of 0.25. After 12 h post-inoculation (hpi), cells were harvested on slides by cytospinning at 600 × *g* at RT for 8 min. Then, the cells on slides were fixed with 100% methanol for 10 min at −20 °C. To visualize PRRSV infection, a double IF staining was performed. Cells were stained for 1 h at 37 °C with mouse mAb against PRRSV nucleocapsid protein (13E2) [32] in combination with one of the following mAbs: mouse mAb against porcine CD163 (2A10), mouse mAb against porcine Sn (41D3), mouse mAb against human cytokeratin (AE1/AE3) or mouse mAb against porcine vimentin (V9) (Table 1). Subsequently, cells were washed and incubated for 1 h at 37 °C with goat anti-mouse IgG2a Alexa Fluor 594 (1:500, Invitrogen) and goat anti-mouse IgG1 FITC. After two further washings, nuclei were counterstained with Hoechst 33342. Total number of cells, single positive cells, and double-positive cells were counted by confocal microscopy and calculated as percentage.

### Statistical analysis

All data were expressed as mean ± standard deviation (SD) from three independent experiments. Statistical analysis was performed with GraphPad Prism statistical software package version 8.0 (GraphPad, San Diego, CA, USA). Differences between sample groups were analyzed using multiple-way analysis of variance (ANOVA) followed by Tukey’s post hoc test. *p* value of < 0.05 was considered significant.

## Results

### Distribution and quantification of CD163 positive cells in the lamina propria of the porcine nasal mucosa

**Figure 2:**
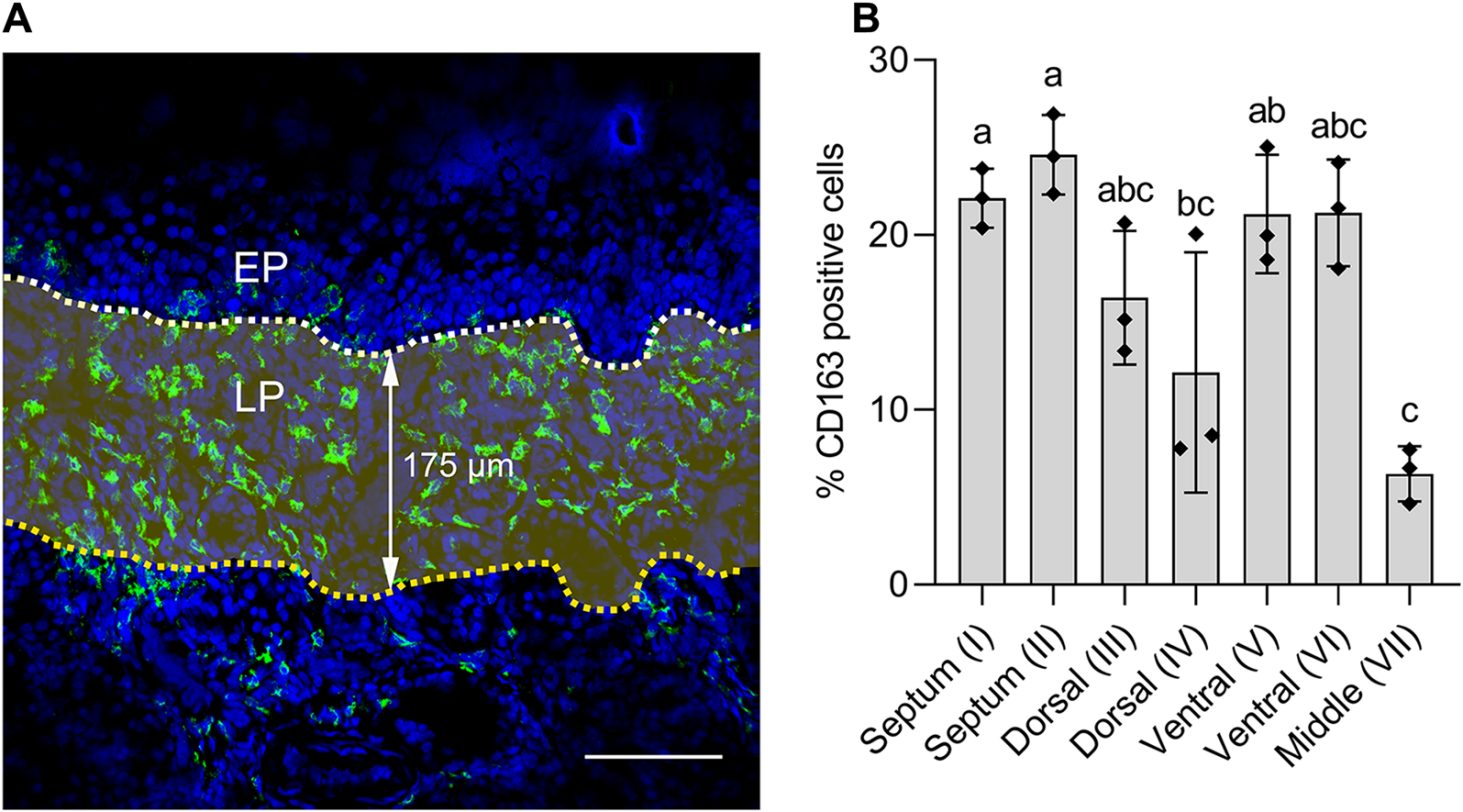
**Quantification of CD163 positive cells in the lamina propria of the porcine nasal mucosa. A** The highlighted area shows where the total number of cells and CD163^+^ cells were counted. Pictures, 175 µm in-depth, were taken under the nasal epithelium using a ×10 ocular lens and ×63 objective. The upper white line indicates the border between the respiratory epithelium and the lamina propria. **B** Percentage of CD163^+^ cells from different parts of the nasal tissues: (I) anterior nasal septum, (II) posterior nasal septum, (III) medial side of the dorsal nasal turbinate, (IV) lateral side of the dorsal nasal turbinate, (V) medial side of the ventral nasal turbinate, (VI) lateral side of the ventral nasal turbinate, and (VII) middle nasal turbinate. Statistical significance was determined by one-way ANOVA followed by Tukey’s multiple comparison post hoc test. Different letters represent significant differences (*p* < 0.05). All data are expressed as mean value of three experiments ± SD. Scale bar: 100 µm.

A single IF staining was performed to identify the distribution and quantification of CD163 positive cells in the lamina propria of the porcine nasal mucosa. CD163^+^ cells were spread throughout the whole nasal mucosa (Figure 1B). Most of the CD163^+^ cells were located in the lamina propria. In addition, many CD163^+^ cells were identified in between epithelial cells and between the epithelial cells and connective tissue of the lamina propria especially from the nasal septum and the ventral turbinate sections (Figure 1B, panels I and V). In order to quantify CD163^+^ cells in the lamina propria, regions of interest (ROIs) were set in the lamina propria with a depth of 175 µm (Figure 2A). The nasal tissues with most CD163^+^ cells were nasal septum (anterior: 20.6 ± 1.7%, posterior: 20.5 ± 4.8%) and ventral turbinates (medial side: 20.0 ± 3.9%, lateral side: 17.6 ± 4.0%) whereas the percentage of CD163^+^ cells in the middle turbinate was much lower (9.5 ± 4.0%). The percentages of CD163^+^ macrophages in both anterior and posterior sides of the septum were significantly higher compared to the middle turbinate (*p* < 0.01) (Figure 2B). In addition, the medial side of the ventral turbinate showed significantly higher percentage of CD163^+^ cells (*p* < 0.01) than the middle turbinate. Although not statistically significant, the percentage of CD163^+^ macrophages in the lateral side of the ventral turbinate was higher than the middle turbinate (*p* = 0.074) (Figure 2B).

### Sialoadhesin (Sn) expression in CD163^−^ and CD163^+^ cells in the porcine nasal mucosa and submucosa

**Figure 3:**
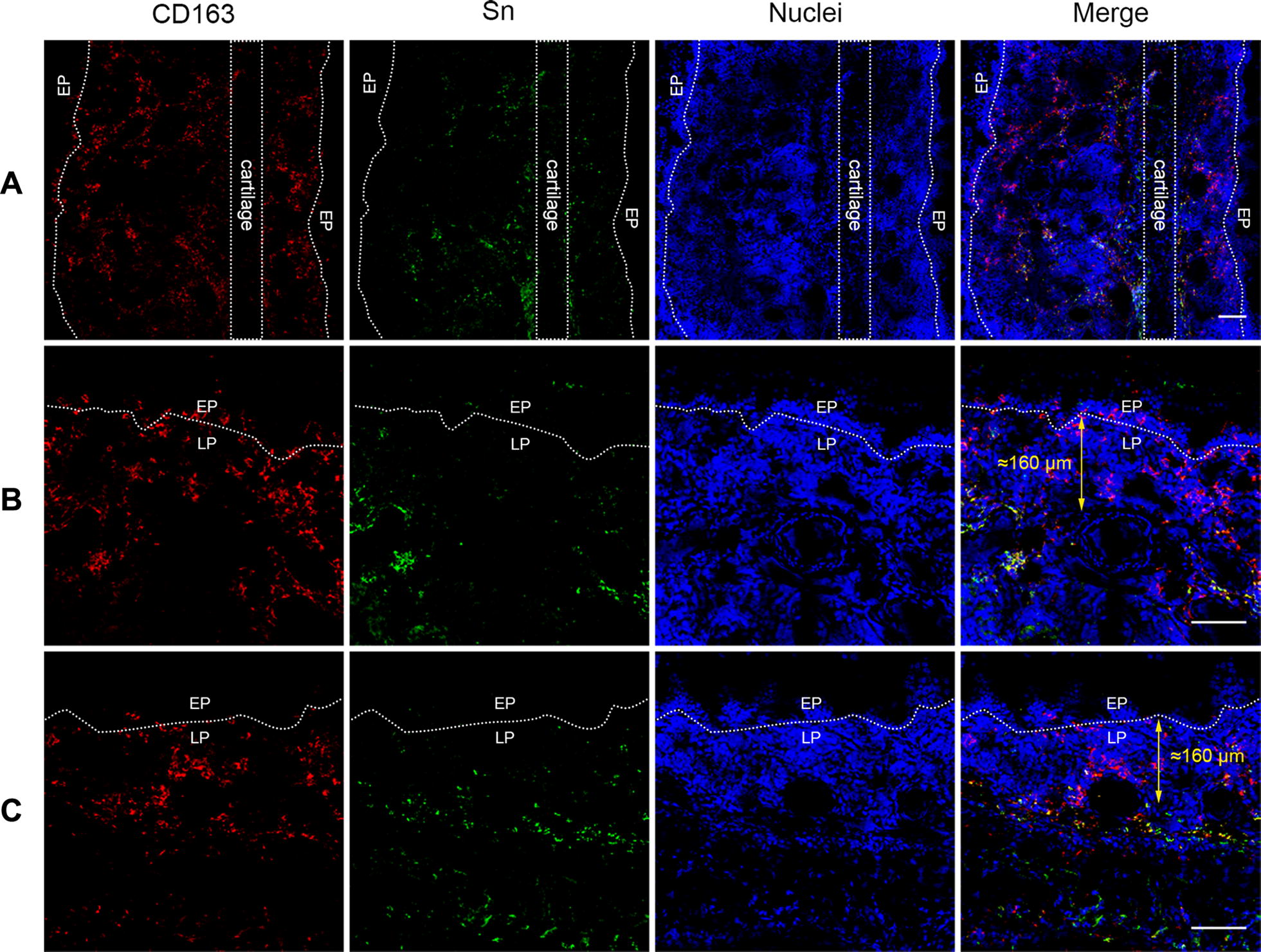
**Sialoadhesin expression in CD163**^**+**^**cells in the ventral nasal turbinate.** Sections of the ventral nasal turbinate were subjected to a double immunofluorescent staining analysis for CD163 and sialoadhesin. **A** Whole ventral nasal turbinate including cartilage, **B** medial side of the ventral nasal turbinate, and **C** lateral side of the ventral nasal turbinate. *EP* epithelium, *LP* lamina propria. Scale bar: 100 µm.

Sn expression in CD163^−^ and CD163^+^ cells was identified by a double IF staining against Sn and CD163 (Figure 3). In both sides of the ventral turbinates, CD163^+^Sn^−^ cells were mainly located in the area of approximately 160 µm underneath the epithelium in the upper lamina propria while CD163^+^Sn^+^ cells were predominant in the connective tissue underneath 160 µm, close to the cartilage. We propose to call these CD163^+^Sn^−^ macrophages “nasal surface macrophages” based on their Sn negative-characteristics and their location in the nasal mucosa. Interestingly, many CD163^−^Sn^+^ cells were observed in the submucosa close to the cartilage.

Additional triple IF staining against CD163, Sn and several macrophage markers showed that 25.2%, 19.3% and 3.4% of the CD163^+^Sn^−^ macrophages were CD1c, MHCII and CD14 positive, respectively (Additional file 1).

### Isolation of CD163^+^Sn^−^ macrophages from the upper nasal lamina propria by the whole nose digestion system

**Figure 4:**
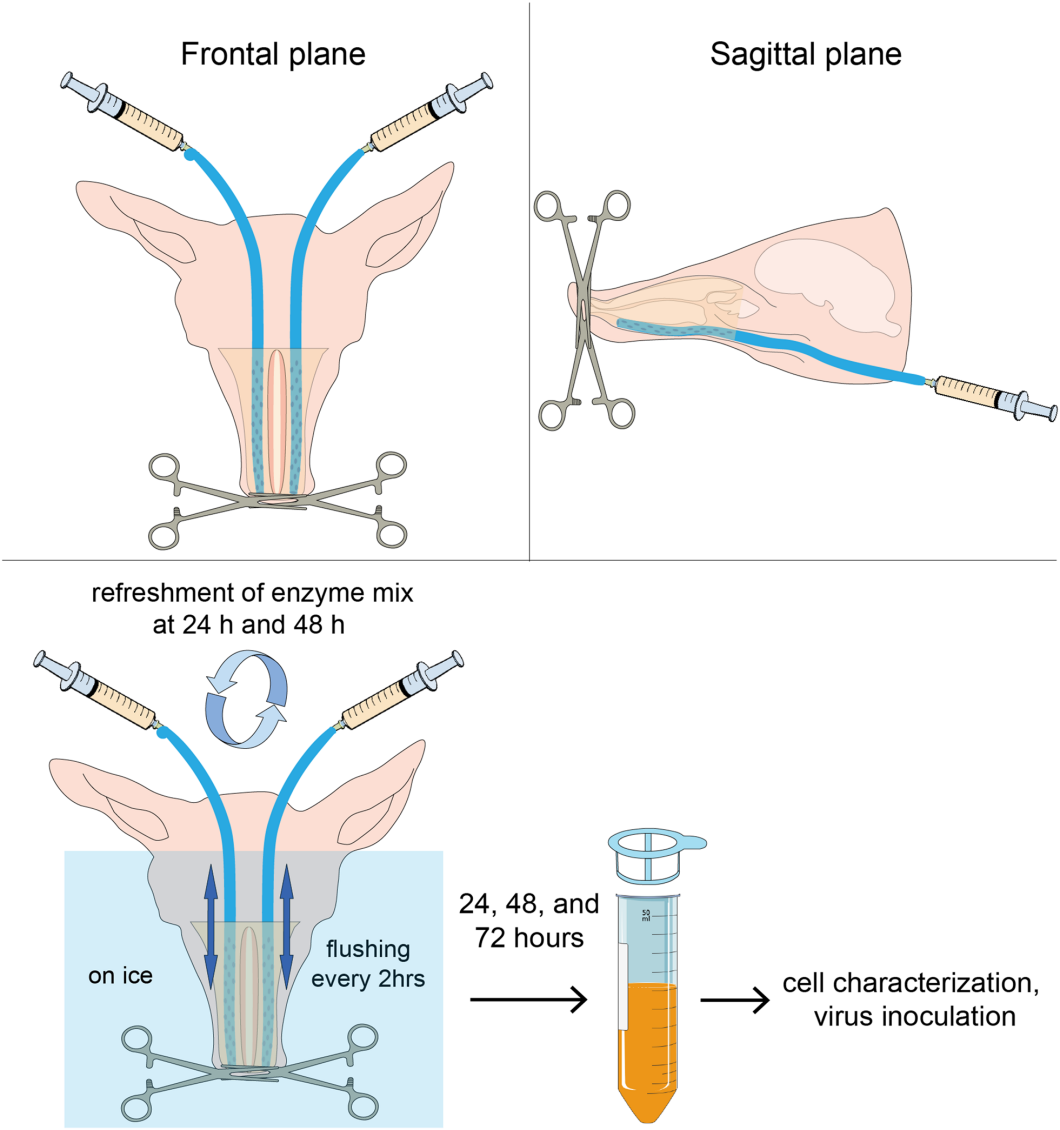
**Schematic representation of the whole nose digestion system.** The top of the figure shows the frontal and sagittal planes of the pig nasal cavity where the tubes (turquoise blue) were inserted. The digestion was performed for 72 h in total. Enzyme mix with dissociated cells was collected and refreshed by a new enzyme mix every 24 h to isolate cells from the lamina propria. Small holes (represented by blue spots) were made at the end of the silicone tubes for flushing the enzyme mix efficiently. The whole nose digestion was performed on ice for a mild enzyme reaction. Cells collected at 24, 48, and 72 h were directly used for cell characterization and susceptibility to PRRSV-1 infection.

**Figure 5:**
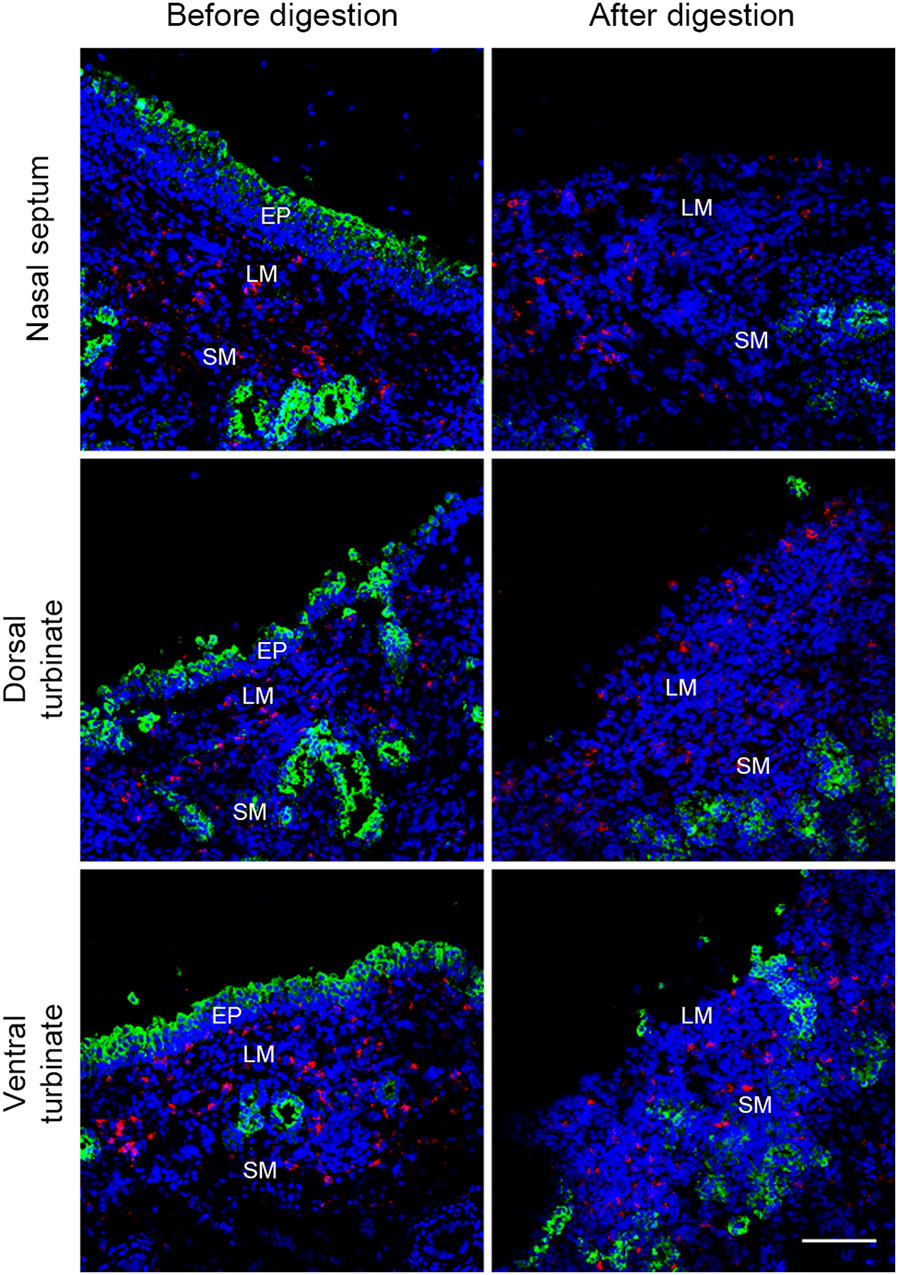
**Double immunofluorescence staining of undigested and digested nasal septum, dorsal turbinate and ventral turbinate before and after digestion.** CD163 (red), cytokeratin (green), and Hoechst (blue). EP: epithelium, LM: lamina propria, SM: submucosa. Scale bar: 100 µm.

**Figure 6:**
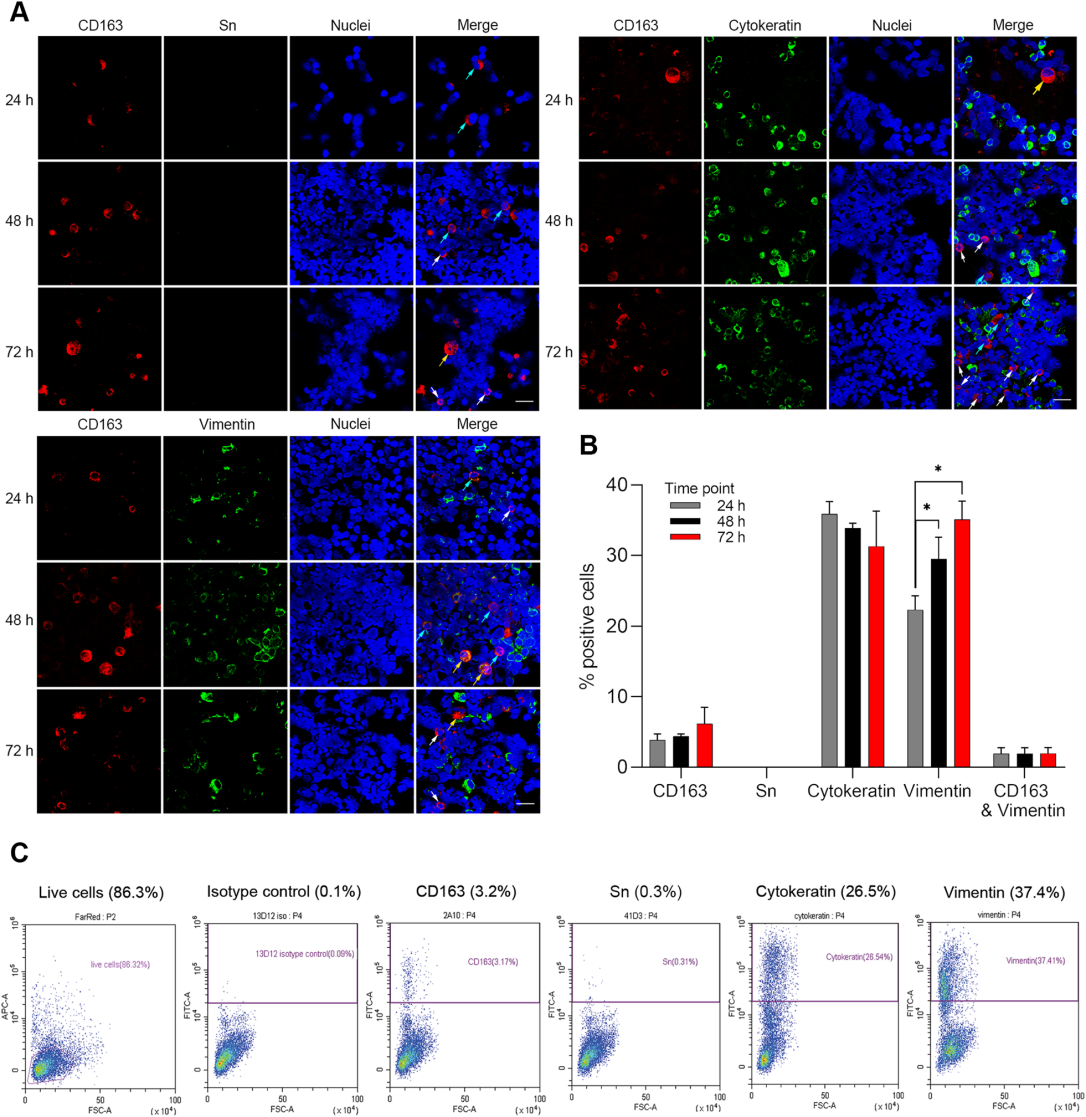
**Characterization of the isolated primary nasal cells at different digestion times. A** Double immunofluorescence staining of CD163 (red) in combination with Sn or vimentin or cytokeratin (green) in cells isolated after 24 h, 48 h, and 72 h digestion. CD163^+^ macrophages of various sizes are indicated with different arrows; yellow arrows (~25 µm), cyan arrows (~16 µm), and white arrows (~10 µm). Scale bar: 25 µm. **B** Percentage of positive cells from each time point are presented. Statistical significance was determined by two-way ANOVA followed by Tukey’s post hoc test (*p < 0.05, **p < 0.01). All data are expressed as mean value of three experiments ± SD. **C** Flow cytometric analysis of the primary nasal cells collected after 72 h digestion.

To isolate CD163^+^Sn^−^ macrophages located in the upper nasal lamina propria (nasal surface macrophages), we developed a whole nose digestion system (Figure 4). Dissociated cells were collected and further characterized. During 3 days, each time after a 24 h digestion period, cells were collected and the nose was replenished with a fresh enzyme mix, allowing to detach cells deeper in the upper lamina propria. A high number of cells (10^7^–10^8^ cells) were collected each day (Additional file 2A). The mean viability was higher than 86% as determined by both trypan blue staining and flow cytometric analysis (Figure 6C and Additional file 2B). By double IF staining against CD163 and cytokeratin on nasal tissues after digestion, it was confirmed that most of the cytokeratin^+^ epithelial cells were removed after 72 h digestion (Figure 5).

### Characterization of the isolated nasal cells

Cells, collected after every 24 h digestion, were cytospinned and stained against CD163 in combination with Sn or cytokeratin or vimentin. CD163^+^ macrophages with various diameters were observed (from 12 to 25 µm diameter) (Figure 6A; yellow, cyan, and white arrows). After 72 h digestion, the majority of cells were identified as vimentin^+^ mesenchymal cells (35.1 ± 2.5%), cytokeratin^+^ epithelial cells (31.2 ± 5.0%) and CD163^+^ macrophages (6.1 ± 2.3%). Only 1.8 ± 0.8% of the cells were CD163^+^vimentin^+^ (Figure 6B). Vimentin^+^ mesenchymal cells significantly increased over digestion times (*p* < 0.05) (Figure 6B). No statistically significant increase or decrease was observed in CD163^+^, Sn^+^, and cytokeratin^+^ cells, collected every 24 h digestion. Although not significant, the percentage of isolated cytokeratin^+^ epithelial cells decreased while isolated CD163^+^ macrophages increased over digestion time (Figure 6B). No Sn^+^ cells were identified by IF staining (Figures 6A, B). In comparison with the IF staining result, flow cytometric analysis of the primary nasal cells collected after 72 h digestion showed a similar percentage of vimentin^+^ cells (37.4%). However, the percentage of CD163^+^ cells and cytokeratin^+^ were somewhat lower (3.2% and 26.5%, respectively) than quantified on the confocal microscopy (Figure 6C). The percentage of Sn^+^ cells (0.3%) was not significantly different from the percentage detected in the isotype control (0.1%) (Figure 6C).

### PRRSV-1, subtype 3 Lena replicates better in the nasal surface macrophages than subtype 1 LV

**Figure 7:**
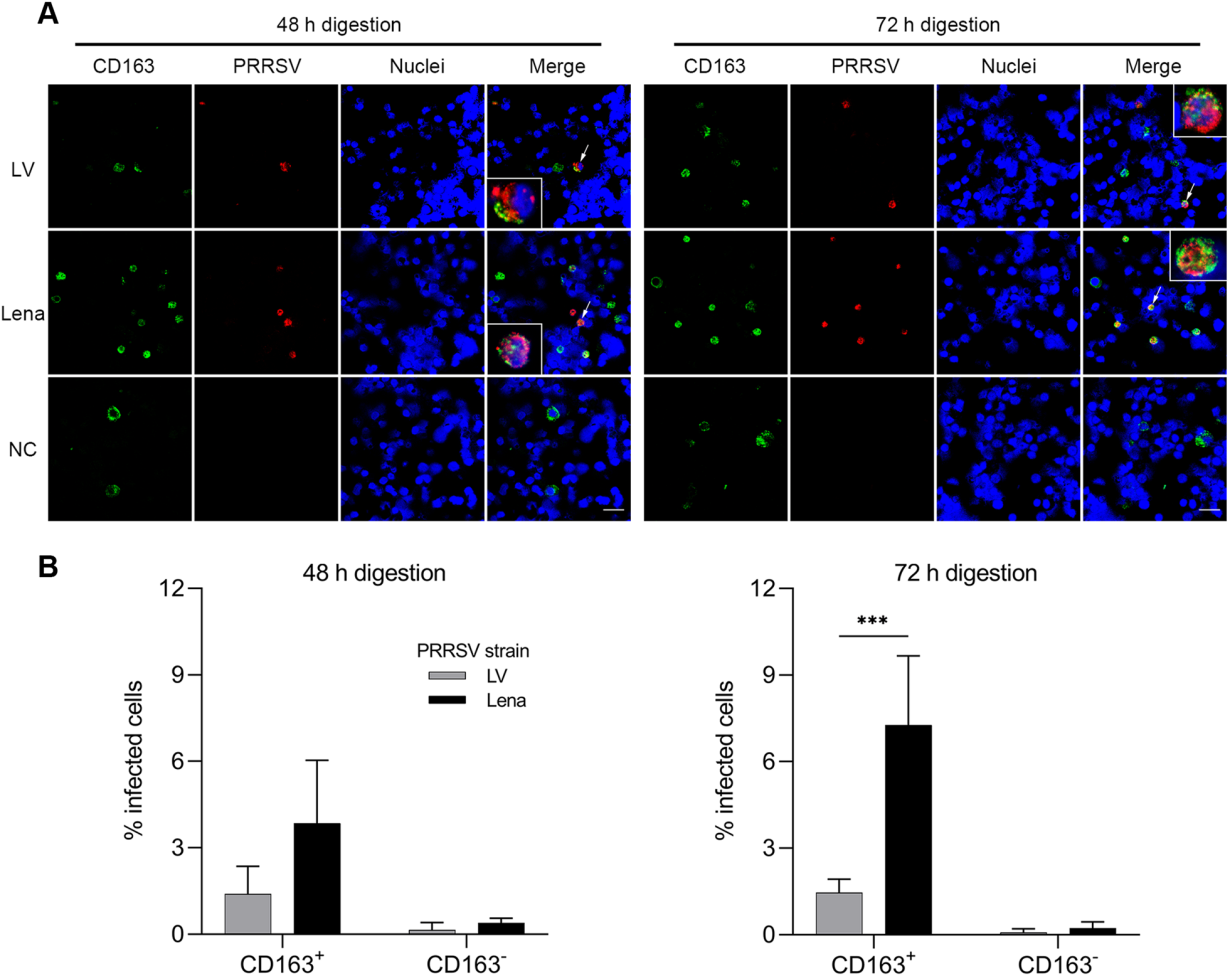
**PRRSV-1 Lena subtype 3 replicates better than LV subtype 1 in nasal surface macrophages.** Primary nasal cells isolated after 48 h and 72 h digestion were inoculated with LV and Lena. **A** Cells were co-immunostained for PRRSV N-protein (red) and CD163 (green) at 12 hpi. Scale bar: 25 µm. Small boxes in the IF pictures represent zoomed pictures of the infected CD163^+^ cells indicated by arrows. **B** Identification and quantification of PRRSV-1 LV and Lena-infected cells. Statistical significance was determined by two-way ANOVA followed by Tukey’s post hoc test (***p < 0.001). All data are expressed as mean value of three experiments ± SD. All inoculated cells are from the same group used for cell characterization (Figure 5).

Double IF staining against PRRSV nucleocapsid protein and against CD163 or Sn or cytokeratin or vimentin was performed for the identification of PRRSV-susceptible cells isolated from the upper nasal lamina propria (Figure 7A and Additional file 4) and the infected cells were quantified (Figure 7B and Additional file 3). In the cells collected after 48 h digestion, we observed a slightly but not statistically significant higher infection in the Lena-inoculated cells (4.2 ± 2.0%) than with LV-inoculated cells (1.5 ± 0.7%) (*p* = 0.073) and more than 90.4% of the infected cells were CD163^+^ (Figure 7B left and Additional file 3A). After 72 h digestion, the percentage of PRRSV-1 infected cells significantly increased in Lena-inoculated cells (7.4 ± 2.1%) compared to LV-inoculated cells (1.5 ± 0.3%) (*p* < 0.001) and more than 95% of infected cells were CD163^+^ (Figure 7B right and Additional file 3B). In addition, both infected CD163^+^ and CD163^−^ cells were identified as negative for cytokeratin and vimentin as well as for Sn (Additional file 4). Taken together, these data demonstrate that the isolated nasal surface macrophages are susceptible to both PRRSV-1 LV and Lena strains, but Lena shows a much higher infection than LV. The majority of infected nasal surface macrophages were characterized as CD163^+^/Sn^−^/cytokeratin^−^/vimentin^−^.

### CD163 expression was increased upon inoculation with PRRSV-1 Lena

**Figure 8:**
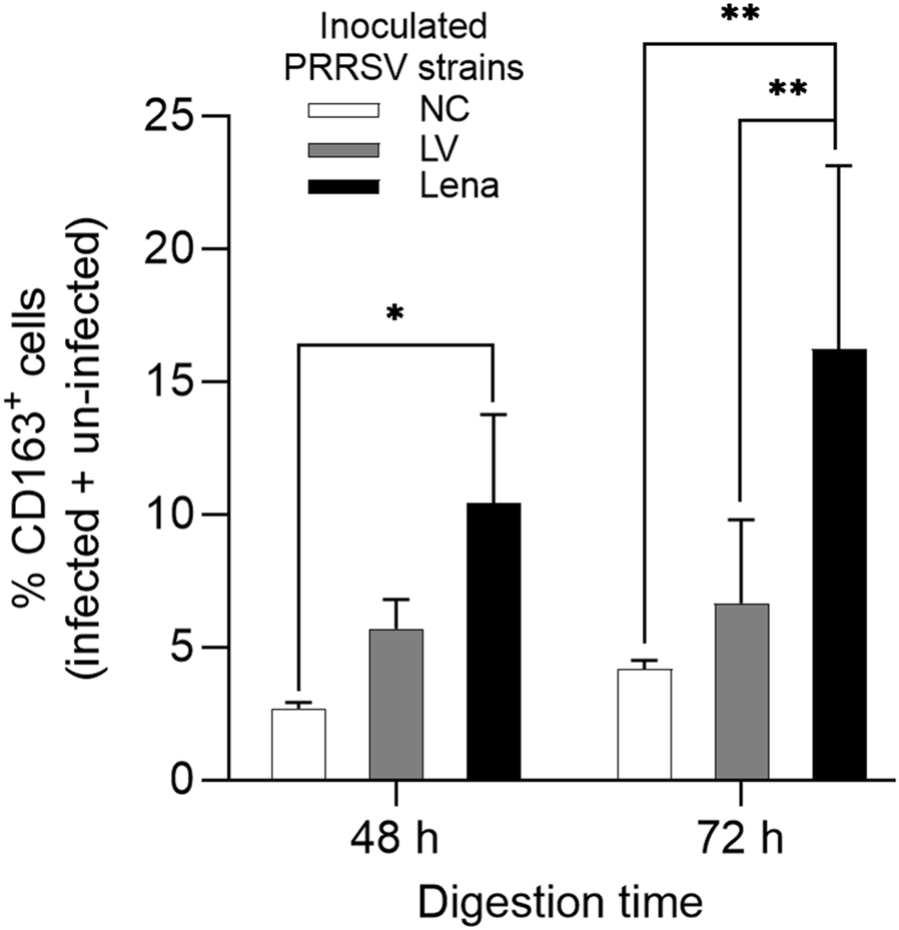
**Increased CD163 expression upon PRRSV-1 Lena inoculation.** Quantification of total CD163^+^ cells from the mock, LV and Lena-inoculated groups. NC: mock-inoculated group. Statistical significance was determined by two-way ANOVA followed by Tukey’s post hoc test (*p < 0.05, **p < 0.01). All data are expressed as mean value of three experiments ± SD. Cells used in this experiment are from the same group used for cell characterization (Figure 5).

During the PRRSV-1 inoculation experiments, we also quantified both infected and non-infected CD163^+^ cells. Interestingly, the percentage of CD163 expressing cells significantly increased in Lena-inoculated cells compared to both LV-inoculated cells (72 h digestion: *p* < 0.05) and mock-inoculated group (48 h digestion: *p* < 0.05 and 72 h digestion: *p* < 0.01) (Figure 8). No statistically significant increase was observed between the mock-inoculated group and the LV-inoculated group. This suggests that CD163 expression was strongly induced by the Lena-inoculation but not by the LV-inoculation.

## Discussion

Transmission of PRRSV can occur in various ways including physical contact, contaminated fomites and/or airborne inhalation [6]. The nasal mucosa is not only a port for virus entry of the host but also the site where the virus is produced and shed. The airborne route of transmission is favorable for highly pathogenic PRRSV strains because of their strong ability to replicate in the nasal mucosa. Previously, Sn and CD163 were considered as major entry mediators for PRRSV infection in PAM [23]. However, recent studies showed that Sn knockout pigs are still susceptible to PRRSV-2 and newly emerging PRRSV strains have a wider cell tropism and that they are capable of infecting CD163^+^Sn^−^ cells in the nasal mucosa [11, 25, 26]. This suggests that some PRRSV strains use an alternative receptor instead of Sn. The effort to find a new putative mediator led to other members of the Siglec family. Xie et al. demonstrated that siglec-10 mediates PRRS viral entry and that the non-permissive cell line PK-15 was even more susceptible to certain PRRSV-1 and PRRSV-2 strains upon co-expression of CD163 and siglec-10 compared to co-expression of CD163 and Sn [13, 14]. However, siglec-10 is only expressed in cells of porcine lymphoid tissues and not in nasal macrophages. In order to isolate the nasal CD163^+^Sn^−^ macrophages for further in vitro research, a new isolation technology was established in the present study.

We first examined the distribution and quantity of the CD163^+^ macrophages in the nasal tissues. Anatomically, the long-narrow porcine nose comprises of a septum and three turbinates; dorsal, middle, and ventral which serve different functions. They guide the inhaled air through the nose allowing it to be filtered, humidified and warmed up (Figure 1A). The spiral structure of turbinates gives the nasal mucosa a larger surface, increasing the chance to capture pathogens by the mucus covering the epithelium. Monocytic cells in the mucosal epithelia interact with each other for capturing, destroying and processing antigens to T-cells [33]. Our study showed that the CD163^+^ cells were most concentrated in the lamina propria of the nasal tissues. Comparing the different parts of the nose, the nasal septum and ventral turbinates contained statistically more CD163^+^ cells compared to the middle and dorsal turbinates. Furthermore, a large number of CD163^+^ cells were exclusively observed in the epithelial cell layer of these two tissues. The larger number of CD163^+^ cells may be related to the fact that these regions are the first contact regions for the incoming air and are continuously flowed with air. The middle and dorsal turbinates are mainly flowed with air during vigorous breathing. Moreover, a double immunofluorescence staining against CD163 and Sn on the same frozen tissues revealed that CD163^+^Sn^−^ cells are located in the area of 160 µm depth of the lamina propria while CD163^+^Sn^+^ cells were more abundantly distributed in deeper connective tissues (submucosa). In addition, CD163^−^Sn^+^ cells were identified in the submucosa close to the cartilage. The different localization of macrophages in non-porcine intestines, epidermis, and hair follicles has been reported before [34–36]. Asano et al. [34] demonstrated that murine intestinal Sn^−^ phagocytes are located near the intestinal epithelium while Sn^+^ macrophages are more localized in deeper tissues. Localization of our nasal CD163^+^ cells by Sn expression status was in agreement with this study. These Sn^−^ macrophages are well positioned to combat pathogens as soon as they enter the respiratory and intestinal mucosae. Future work will be done on the anti-pathogen defense mechanisms in these cells. Why the upper respiratory tract surface macrophages are Sn-negative and alveolar and interstitial lung macrophages are Sn-positive is not clear at the moment. During evolution, Sn has been specifically expressed in the lung macrophages. Porcine macrophage markers are poorly investigated compared with human and mice. Additional triple IF staining against several macrophage makers together with CD163 and Sn on the ventral turbinate section showed that CD163^+^Sn^−^ cells located in the lamina propria are phenotypically diverse (Additional file 1) [37]. For a better characterization, CD80 and macrophage mannose receptor could be used as M1/M2 macrophage markers. Nasal macrophages have been rarely studied in human or mice as well as pigs. Since most of the studies on porcine macrophages are focused on PAM or monocyte-derived macrophages, an approach with various immune cell markers is necessary for studying macrophages isolated from other tissues [38–40].

Next, based on the identified location of the nasal CD163^+^Sn^−^ cells (designated as nasal surface macrophages), we developed a system for the isolation of this new cell population (Figure 4). In the present study, a combination of collagenase IV and pronase was used for the superficial digestion of the upper nasal tissue. The nasal mucosa consists of the epithelium which is supported by the basement membrane and the lamina propria. Collagen is the major component of the basement membrane and the lamina propria [41]. Pronase separates epithelial cells from the lamina propria during treatment of 4 °C [42, 43] and collagenase type IV is designed to be especially low in tryptic activity to limit damage to membrane proteins and receptors but with normal to above normal collagenase activity [44]. In our system, the nasal epithelium and the basement membrane were effectively dissociated by these two enzymes mixture, without damaging the nasal cells. In addition, applying the enzyme into the whole nasal mucosa without separating them from the cartilage and bone allowed cells to be dissociated sequentially from the epithelium to the lamina propria. Furthermore, excessive digestion into the submucosa was mitigated by increasing the reaction time at low temperature and refreshment with a new enzyme mix every 24 h increased the digestion efficiency.

The isolated nasal cell population was then characterized by the detection of cell type markers of epithelial and mesenchymal cells and macrophages. The lamina propria is a thin layer of connective tissue composed of various mesenchymal cell types. Our results have shown that 72 h of digestion was sufficient for isolating our cells of interest located in the upper lamina propria. The proportion of isolated vimentin^+^ mesenchymal cells significantly increased over time. At 72 h of digestion, the number of vimentin^+^ cells was even higher than the number of cytokeratin^+^ cells. After 72 h of digestion, the cytokeratin^+^ epithelial layer was completely removed, demonstrating the efficiency of the performed digestion. Although Sn^+^ cells could not be identified by confocal microscopy, 0.3% Sn^+^ macrophages were identified by flow cytometry. However, this percentage did not differ significantly from the percentage of positive cells in the isotype controls (0.1%). Sn negative cells are mainly located in the upper region of the lamina propria and the enzyme mixture could easily reach the region underneath the basement membrane, where most CD163^+^Sn^−^ cells are localized. Therefore, as expected most if not all isolated macrophages were Sn^−^. Taken together, all CD163^+^ cells of the isolated primary cells were considered to be Sn^−^, indicating that this digestion method is very successful to preferentially isolate nasal surface macrophages.

A previous study using nasal explants demonstrated for PRRSV-1 subtype 3 Lena that most of the infected cells are CD163^+^Sn^−^ and located within or in the proximity of the epithelium in the nasal mucosa [11]. To investigate the cell tropism of our primary nasal cells collected by the whole nose digestion system, isolated cells were inoculated with PRRSV-1 subtype 1 LV and subtype 3 Lena. Here, we showed that isolated nasal surface macrophages are susceptible to both PRRSV-1 LV and Lena, but that Lena has a much stronger tropism for this cell type. At 12 hpi, Lena was found to infect 2.8 times more cells than LV in the nasal cells collected after 48 h digestion. With the cells collected after 72 h digestion, Lena infected 4.9 times more cells than LV (*p *< 0.01). Ninety percent of the infected cell type was CD163^+^ in the 48 h cells and this percentage increased to 95% in the 72 h group. Infected cells were further characterized as Sn^−^, cytokeratin^−^, and vimentin^−^. No PRRSV^+^Sn^+^ cells were observed by confocal microscopy, which is logical since Sn^+^ macrophages were absent. Our results indicate that the attachment and entry of PRRSV-1, especially subtype 3 Lena is mediated by a receptor different from Sn. In our study, PRRSV infection was also detected in CD163^−^ cells isolated from the upper lamina propria. This is in contrast with the previous studies demonstrating that CD163 is an indispensable PRRSV infection mediator. In vivo, CD163 gene-edited pigs or CD163 knock out pigs were protected from PRRSV infection [16, 20, 21]. However, there are some previous studies that are consistent with our results. Frydas et al. [11] also observed Lena strain infection in CD163^−^Sn^−^ cells in the nasal mucosa explants and Doeschl-Wilson et al. [45] found that the population of infected CD163^−^ PAM increased over incubation time. Also, Li et al. [46] recently demonstrated PRRSV infection in CD163^−^, CD163^lo^ and CD163^hi^ cells. This means that in vitro, CD163 positive cells may still be infected with certain PRRSV strains.

The percentage of CD163^+^ cells (both infected and non-infected) was largely higher in the Lena-inoculated group than both LV-inoculated group (*p* < 0.05) and mock-inoculated group (*p* < 0.01). This is in agreement with a previous study that has shown that CD163 expression in lung cells was up-regulated after PRRSV-2 infection [47]. IL-10 is known as a strong inducer of CD163 expression both in vitro and in vivo [48, 49]. Since it is known that certain PRRSV strains induce IL-10 production in PBMCs, mature DC, bronchoalveolar macrophages and PAM [50–54], it is highly possible that this cytokine caused CD163 upregulation in the nasal cells.

In summary, the present study is the first to provide a cell isolation system from the whole pig nose without mechanical tissue separation. Our enzyme digestion system successfully isolated CD163^+^Sn^−^ cells. Our results demonstrated distinguishing features of nasal surface macrophages. They are (i) Sn negative, (ii) localized in the upper lamina propria, and (iii) show higher susceptibility to Lena compared to LV. The alternative PRRSV binding and internalization receptor for these nasal surface macrophages remains unknown. Our new cell isolation system forms the basis for future research on the molecular pathogenesis of PRRSV in the nose and for further functional and phenotypical analysis of this new population (nasal surface macrophages).

## Supplementary information

**Additional file 1. Characterization of the CD163**^**+**^**Sn**^**-**^**cells by a triple IF staining against macrophages markers (red, CD1c: green), Sn (green or red) and CD163 (magenta).** EP: epithelium, LP: lamina propria and SM: submucosa. Scale bar: 100 µm.

**Additional file 2.** (A) Total number of collected primary nasal cells at each digestion time point and (B) their viability determined by the trypan blue staining. SD: standard deviation.

**Additional file 3.** (A) Percentage of PRRSV-1 LV infected cells and (B) PRRSV-1 Lena infected cells counted after double immunofluorescence staining. Values represent percentage of CD163 positive and CD163 negative cells in the whole population of infected cells (CD163^−^+ CD163^+^ = 100%).

**Additional file 4. PRRSV-1 infected cells are negative for Sn, cytokeratin and vimentin.** Primary nasal cells isolated after 48 h and 72 h digestion were inoculated with PRRSV-1 LV or Lena. Cells were co-immunostained for PRRSV N-protein (red) and markers for Sn, cytokeratin and vimentin (green) at 12 hpi. Scale bar: 25 µm.

## Abbreviations

ANOVA: analysis of variance
DC: dendritic cells
DPBS: Dulbecco’s phosphate-buffered saline
EDTA: ethylene diamine tetra-acetic acid
FCS: fetal calf serum
FITC: fluorescein isothiocyanate
hpi: hour post-inoculation
IF: immunofluorescence
mAb: monoclonal antibody
MOI: multiplicity of infection
MYH9: non-muscle myosin heavy chain 9
pAb: polyclonal antibody
PAM: porcine alveolar macrophages
PBMC: peripheral blood mononuclear cells
PBS: phosphate-buffered saline
PRRSV: porcine reproductive and respiratory syndrome virus
PRV gD: pseudorabies virus gD
RT: room temperature
SD: standard deviation
Sn: sialoadhesin

## References

[1] Nieuwenhuis N, Duinhof TF, van Nes A (2012). Economic analysis of outbreaks of porcine reproductive and respiratory syndrome virus in nine sow herds. Vet Rec.

[2] Mettenleiter TC, Sobrino F (2008). Animal viruses: molecular biology.

[3] Siddell SG, Walker PJ, Lefkowitz EJ, Mushegian AR, Adams MJ, Dutilh BE, Gorbalenya AE, Harrach B, Harrison RL, Junglen S, Knowles NJ, Kropinski AM, Krupovic M, Kuhn JH, Nibert M, Rubino L, Sabanadzovic S, Sanfacon H, Simmonds P, Varsani A, Zerbini FM, Davison AJ (2019). Additional changes to taxonomy ratified in a special vote by the International Committee on Taxonomy of Viruses (October 2018). Arch Virol.

[4] Williamson SF, Frossard JP, Thomson J (2018). PRRS diagnoses in Great Britain 2016/17. Vet Rec.

[5] Karniychuk UU, Geldhof M, Vanhee M, Van Doorsselaere J, Saveleva TA, Nauwynck HJ (2010). Pathogenesis and antigenic characterization of a new East European subtype 3 porcine reproductive and respiratory syndrome virus isolate. BMC Vet Res.

[6] Pileri E, Mateu E (2016). Review on the transmission porcine reproductive and respiratory syndrome virus between pigs and farms and impact on vaccination. Vet Res.

[7] Takaki H, Ichimiya S, Matsumoto M, Seya T (2018). Mucosal immune response in nasal-associated lymphoid tissue upon intranasal administration by adjuvants. J Innate Immun.

[8] Russell MW, Mestecky J, Strober W, Lambrecht BN, Kelsall BL, Cheroutre H, Russell MW, Strober W, Lambrecht BN, Kelsall BL, Cheroutre H (2015). Chapter 1—Overview: the mucosal immune system. Mucosal immunology.

[9] Steukers L, Glorieux S, Vandekerckhove AP, Favoreel HW, Nauwynck HJ (2012). Diverse microbial interactions with the basement membrane barrier. Trends Microbiol.

[10] Vareille M, Kieninger E, Edwards MR, Regamey N (2011). The airway epithelium: soldier in the fight against respiratory viruses. Clin Microbiol Rev.

[11] Frydas IS, Verbeeck M, Cao J, Nauwynck HJ (2013). Replication characteristics of porcine reproductive and respiratory syndrome virus (PRRSV) European subtype 1 (Lelystad) and subtype 3 (Lena) strains in nasal mucosa and cells of the monocytic lineage: indications for the use of new receptors of PRRSV (Lena). Vet Res.

[12] Gao J, Xiao S, Xiao Y, Wang X, Zhang C, Zhao Q, Nan Y, Huang B, Liu H, Liu N, Lv J, Du T, Sun Y, Mu Y, Wang G, Syed SF, Zhang G, Hiscox JA, Goodfellow I, Zhou EM (2016). MYH9 is an essential factor for porcine reproductive and respiratory syndrome virus infection. Sci Rep.

[13] Xie J, Christiaens I, Yang B, Breedam WV, Cui T, Nauwynck HJ (2017). Molecular cloning of porcine Siglec-3, Siglec-5 and Siglec-10, and identification of Siglec-10 as an alternative receptor for porcine reproductive and respiratory syndrome virus (PRRSV). J Gen Virol.

[14] Xie J, Christiaens I, Yang B, Trus I, Devriendt B, Cui T, Wei R, Nauwynck HJ (2018). Preferential use of Siglec-1 or Siglec-10 by type 1 and type 2 PRRSV strains to infect PK15(S1-CD163) and PK15(S10-CD163) cells. Vet Res.

[15] Zhang Q, Yoo D (2015). PRRS virus receptors and their role for pathogenesis. Vet Microbiol.

[16] Burkard C, Opriessnig T, Mileham AJ, Stadejek T, Ait-Ali T, Lillico SG, Whitelaw CBA, Archibald AL (2018). Pigs lacking the scavenger receptor cysteine-rich domain 5 of CD163 are resistant to porcine reproductive and respiratory syndrome virus 1 infection. J Virol.

[17] Calvert JG, Slade DE, Shields SL, Jolie R, Mannan RM, Ankenbauer RG, Welch SK (2007). CD163 expression confers susceptibility to porcine reproductive and respiratory syndrome viruses. J Virol.

[18] Delrue I, Van Gorp H, Van Doorsselaere J, Delputte PL, Nauwynck HJ (2010). Susceptible cell lines for the production of porcine reproductive and respiratory syndrome virus by stable transfection of sialoadhesin and CD163. BMC Biotechnol.

[19] Li L, Wu C, Hou G, Xue B, Xie S, Zhao Q, Nan Y, Zhang G, Zhou EM (2017). Generation of murine macrophage-derived cell lines expressing porcine CD163 that support porcine reproductive and respiratory syndrome virus infection. BMC Biotechnol.

[20] Whitworth KM, Rowland RR, Ewen CL, Trible BR, Kerrigan MA, Cino-Ozuna AG, Samuel MS, Lightner JE, McLaren DG, Mileham AJ, Wells KD, Prather RS (2016). Gene-edited pigs are protected from porcine reproductive and respiratory syndrome virus. Nat Biotechnol.

[21] Yang H, Zhang J, Zhang X, Shi J, Pan Y, Zhou R, Li G, Li Z, Cai G, Wu Z (2018). CD163 knockout pigs are fully resistant to highly pathogenic porcine reproductive and respiratory syndrome virus. Antiviral Res.

[22] Van Breedam W, Delputte PL, Van Gorp H, Misinzo G, Vanderheijden N, Duan X, Nauwynck HJ (2010). Porcine reproductive and respiratory syndrome virus entry into the porcine macrophage. J Gen Virol.

[23] Van Gorp H, Van Breedam W, Delputte PL, Nauwynck HJ (2008). Sialoadhesin and CD163 join forces during entry of the porcine reproductive and respiratory syndrome virus. J Gen Virol.

[24] Vanderheijden N, Delputte PL, Favoreel HW, Vandekerckhove J, Van Damme J, van Woensel PA, Nauwynck HJ (2003). Involvement of sialoadhesin in entry of porcine reproductive and respiratory syndrome virus into porcine alveolar macrophages. J Virol.

[25] Frydas IS, Trus I, Kvisgaard LK, Bonckaert C, Reddy VR, Li Y, Larsen LE, Nauwynck HJ (2015). Different clinical, virological, serological and tissue tropism outcomes of two new and one old Belgian type 1 subtype 1 porcine reproductive and respiratory virus (PRRSV) isolates. Vet Res.

[26] Prather RS, Rowland RR, Ewen C, Trible B, Kerrigan M, Bawa B, Teson JM, Mao J, Lee K, Samuel MS, Whitworth KM, Murphy CN, Egen T, Green JA (2013). An intact sialoadhesin (Sn/SIGLEC1/CD169) is not required for attachment/internalization of the porcine reproductive and respiratory syndrome virus. J Virol.

[27] Duan X, Nauwynck HJ, Favoreel H, Pensaert MB (1998). Porcine reproductive and respiratory syndrome virus infection of alveolar macrophages can be blocked by monoclonal antibodies against cell surface antigens. Adv Exp Med Biol.

[28] Thacker E, Summerfield A, McCullough K, Ezquerra A, Dominguez J, Alonso F, Lunney J, Sinkora J, Haverson K (2001). Summary of workshop findings for porcine myelomonocytic markers. Vet Immunol Immunopathol.

[29] De Schryver M, Van Gorp H, Hoebeke I, De Maeyer B, Ooms K, Pintelon I, Maes LJ, Cos P, Nauwynck HJ, Delputte PL (2016). Development and characterization of new species cross-reactive anti-sialoadhesin monoclonal antibodies. Antibodies.

[30] Saha D, Huang L, Bussalleu E, Lefebvre DJ, Fort M, Van Doorsselaere J, Nauwynck HJ (2012). Antigenic subtyping and epitopes’ competition analysis of porcine circovirus type 2 using monoclonal antibodies. Vet Microbiol.

[31] Nauwynck HJ, Pensaert MB (1995). Effect of specific antibodies on the cell-associated spread of pseudorabies virus in monolayers of different cell types. Arch Virol.

[32] Van Breedam W, Costers S, Vanhee M, Gagnon CA, Rodriguez-Gomez IM, Geldhof M, Verbeeck M, Van Doorsselaere J, Karniychuk U, Nauwynck HJ (2011). Porcine reproductive and respiratory syndrome virus (PRRSV)-specific mAbs: supporting diagnostics and providing new insights into the antigenic properties of the virus. Vet Immunol Immunopathol.

[33] Steinman RM, Banchereau J (2007). Taking dendritic cells into medicine. Nature.

[34] Asano K, Takahashi N, Ushiki M, Monya M, Aihara F, Kuboki E, Moriyama S, Iida M, Kitamura H, Qiu CH, Watanabe T, Tanaka M (2015). Intestinal CD169(+) macrophages initiate mucosal inflammation by secreting CCL8 that recruits inflammatory monocytes. Nat Commun.

[35] Owen RL, Allen CL, Stevens DP (1981). Phagocytosis of Giardia muris by macrophages in Peyer’s patch epithelium in mice. Infect Immun.

[36] van den Oord JJ, de Wolf-Peeters C (1994). Epithelium-lining macrophages in psoriasis. Br J Dermatol.

[37] Chavez-Galan L, Olleros ML, Vesin D, Garcia I (2015). Much more than M1 and M2 macrophages, there are also CD169(+) and TCR(+) macrophages. Front Immunol.

[38] Bordet E, Maisonnasse P, Renson P, Bouguyon E, Crisci E, Tiret M, Descamps D, Bernelin-Cottet C, Urien C, Lefevre F, Jouneau L, Bourry O, Leplat JJ, Schwartz-Cornil I, Bertho N (2018). Porcine alveolar macrophage-like cells are pro-inflammatory pulmonary intravascular macrophages that produce large titers of porcine reproductive and respiratory syndrome virus. Sci Rep.

[39] Piriou-Guzylack L, Salmon H (2008). Membrane markers of the immune cells in swine: an update. Vet Res.

[40] Singleton H, Graham SP, Bodman-Smith KB, Frossard JP, Steinbach F (2016). Establishing porcine monocyte-derived macrophage and dendritic cell systems for studying the interaction with PRRSV-1. Front Microbiol.

[41] Albert B, Johnson A, Lewis J, Raff M, Roberts K, Walter P (2002) Cell junctions, cell adhesion, and the extracellular matrix. In: Molecular biology of the cell, 4th edn. Garland Science, New York, pp xxxiv, 1463, 1486

[42] Werner U, Kissel T (1995). Development of a human nasal epithelial cell culture model and its suitability for transport and metabolism studies under in vitro conditions. Pharm Res.

[43] Werner U, Kissel T (1996). In-vitro cell culture models of the nasal epithelium: a comparative histochemical investigation of their suitability for drug transport studies. Pharm Res.

[44] Autengruber A, Gereke M, Hansen G, Hennig C, Bruder D (2012). Impact of enzymatic tissue disintegration on the level of surface molecule expression and immune cell function. Eur J Microbiol Immunol.

[45] Doeschl-Wilson A, Wilson A, Nielsen J, Nauwynck H, Archibald A, Ait-Ali T (2016). Combining laboratory and mathematical models to infer mechanisms underlying kinetic changes in macrophage susceptibility to an RNA virus. BMC Syst Biol.

[46] Li YL, Darwich L, Mateu E (2018). Characterization of the attachment and infection by Porcine reproductive and respiratory syndrome virus 1 isolates in bone marrow-derived dendritic cells. Vet Microbiol.

[47] Xiao S, Jia J, Mo D, Wang Q, Qin L, He Z, Zhao X, Huang Y, Li A, Yu J, Niu Y, Liu X, Chen Y (2010). Understanding PRRSV infection in porcine lung based on genome-wide transcriptome response identified by deep sequencing. PLoS ONE.

[48] Sulahian TH, Hogger P, Wahner AE, Wardwell K, Goulding NJ, Sorg C, Droste A, Stehling M, Wallace PK, Morganelli PM, Guyre PM (2000). Human monocytes express CD163, which is upregulated by IL-10 and identical to p155. Cytokine.

[49] Villalta SA, Rinaldi C, Deng B, Liu G, Fedor B, Tidball JG (2011). Interleukin-10 reduces the pathology of mdx muscular dystrophy by deactivating M1 macrophages and modulating macrophage phenotype. Hum Mol Genet.

[50] Flores-Mendoza L, Silva-Campa E, Resendiz M, Osorio FA, Hernandez J (2008). Porcine reproductive and respiratory syndrome virus infects mature porcine dendritic cells and up-regulates interleukin-10 production. Clin Vaccine Immunol.

[51] Renson P, Rose N, Le Dimna M, Mahe S, Keranflec’h A, Paboeuf F, Belloc C, Le Potier MF, Bourry O (2017). Dynamic changes in bronchoalveolar macrophages and cytokines during infection of pigs with a highly or low pathogenic genotype 1 PRRSV strain. Vet Res.

[52] Song S, Bi J, Wang D, Fang L, Zhang L, Li F, Chen H, Xiao S (2013). Porcine reproductive and respiratory syndrome virus infection activates IL-10 production through NF-kappaB and p38 MAPK pathways in porcine alveolar macrophages. Dev Comp Immunol.

[53] Suradhat S, Thanawongnuwech R (2003). Upregulation of interleukin-10 gene expression in the leukocytes of pigs infected with porcine reproductive and respiratory syndrome virus. J Gen Virol.

[54] Suradhat S, Thanawongnuwech R, Poovorawan Y (2003). Upregulation of IL-10 gene expression in porcine peripheral blood mononuclear cells by porcine reproductive and respiratory syndrome virus. J Gen Virol.

