# Supplementary figures and images for: Isolation and characterization of a new population of nasal surface macrophages and their susceptibility to PRRSV-1 subtype 1 (LV) and subtype 3 (Lena)
Source: Vet Res. 2020 Feb 24;51:21. doi: 10.1186/s13567-020-00751-7 (PMC7038536; doi:10.1186/s13567-020-00751-7)

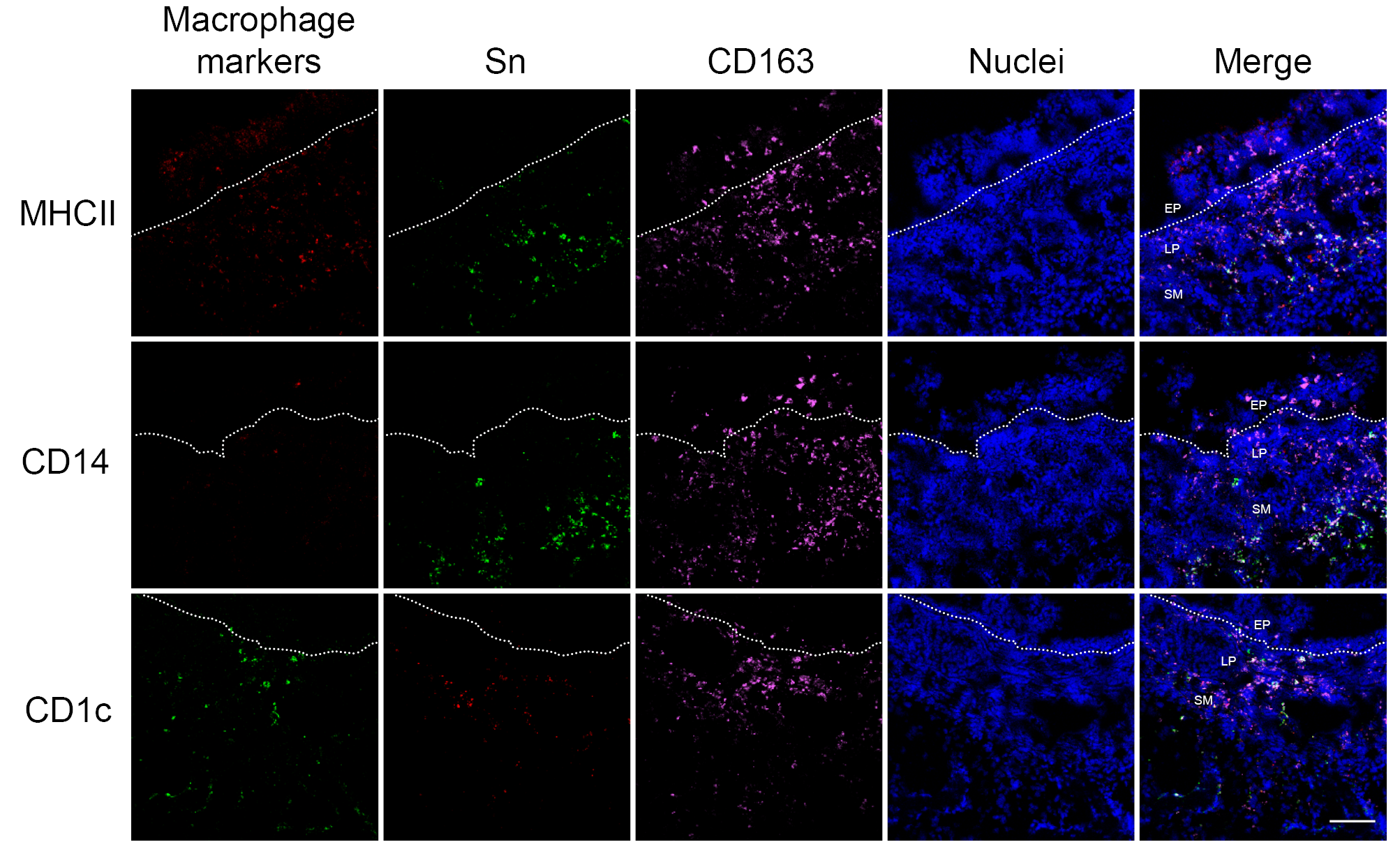

Supplement: Supplementary file 1 — Additional file 1. Characterization of the CD163+Sn-cells by a triple IF staining against macrophages markers (red, CD1c: green), Sn (green or red) and CD163 (magenta). EP: epithelium, LP: lamina propria and SM: submucosa. Scale bar: 100 µm. [file 13567_2020_751_MOESM1_ESM.png]

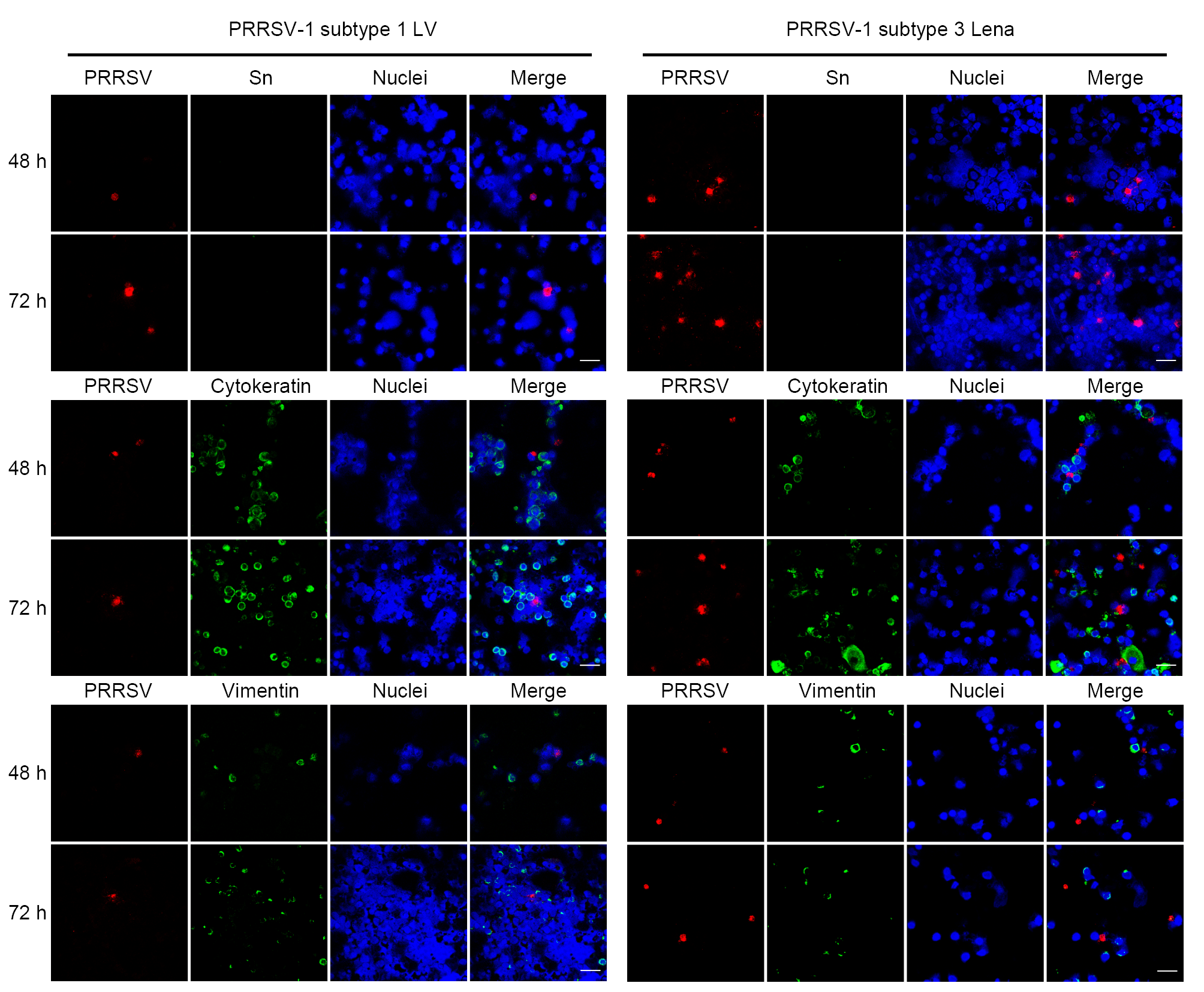

Supplement: Supplementary file 4 — Additional file 4. PRRSV-1 infected cells are negative for Sn, cytokeratin and vimentin. Primary nasal cells isolated after 48 h and 72 h digestion were inoculated with PRRSV-1 LV or Lena. Cells were co-immunostained for PRRSV N-protein (red) and markers for Sn, cytokeratin and vimentin (green) at 12 hpi. Scale bar: 25 µm. [file 13567_2020_751_MOESM4_ESM.png]
