# Supplementary material for: Isolation and characterization of a new population of nasal surface macrophages and their susceptibility to PRRSV-1 subtype 1 (LV) and subtype 3 (Lena)
Source: Vet Res. 2020 Feb 24;51:21. doi: 10.1186/s13567-020-00751-7 (PMC7038536; doi:10.1186/s13567-020-00751-7)
Supplement: Supplementary file 2 — Additional file 2. (A) Total number of collected primary nasal cells at each digestion time point and (B) their viability determined by the trypan blue staining. SD: standard deviation. [file 13567_2020_751_MOESM2_ESM.docx]

(A)

| Digestion time | Total number of collected primary nasal cells | | | |
| --- | --- | --- | --- | --- |
|  | Pig 1 | Pig 2 | Pig 3 | mean ± SD |
| 24 h | 4.6 × 10^7^ | 7.0 × 10^7^ | 2.6 × 10^7^ | 4.7±2.1 × 10^7^ |
| 48 h | 1.2 × 10^8^ | 1.5 × 10^8^ | 7.4 × 10^7^ | 1.1±0.4 × 10^8^ |
| 72 h | 2.5 × 10^8^ | 1.3 × 10^8^ | 1.0 × 10^8^ | 1.6±0.8 × 10^8^ |

(B)

| Digestion time | Cell viability (%) | | | |
| --- | --- | --- | --- | --- |
|  | Pig 1 | Pig 2 | Pig 3 | mean ± SD |
| 24 h | 88 | 94 | 84 | 88.6 ± 5.0 |
| 48 h | 92 | 89 | 86 | 89.0 ± 3.0 |
| 72 h | 87 | 87 | 86 | 86.6 ± 0.5 |
