# Supplementary material for: Isolation and characterization of a new population of nasal surface macrophages and their susceptibility to PRRSV-1 subtype 1 (LV) and subtype 3 (Lena)
Source: Vet Res. 2020 Feb 24;51:21. doi: 10.1186/s13567-020-00751-7 (PMC7038536; doi:10.1186/s13567-020-00751-7)
Supplement: Supplementary file 3 — Additional file 3. (A) Percentage of PRRSV-1 LV infected cells and (B) PRRSV-1 Lena infected cells counted after double immunofluorescence staining. Values represent percentage of CD163 positive and CD163 negative cells in the whole population of infected cells (CD163−+ CD163+ = 100%). [file 13567_2020_751_MOESM3_ESM.docx]

(A)

| Digestion time | % of LV infected cells identified as … | |
| --- | --- | --- |
|  | CD163^+^ | CD163^-^ |
| 48h | 90.4 | 9.6 |
| 72h | 95.0 | 5.0 |

(B)

| Digestion time | % of Lena infected cells identified as … | |
| --- | --- | --- |
|  | CD163^+^ | CD163^-^ |
| 48h | 90.9 | 9.1 |
| 72h | 96.9 | 3.1 |
